# Variability of longitudinal sleep monitoring in amyloid‐negative and amyloid‐positive cognitively unimpaired and mildly impaired older adults

**DOI:** 10.1002/alz.70761

**Published:** 2025-10-08

**Authors:** Taylor J. Pedersen, Ruijin Lu, Cristina Toedebusch, Ashley Hess, Rachel Richardson, Allyson Quigley, Carling G. Robinson, John C. Morris, David M. Holtzman, Chengjie Xiong, Brian A. Gordon, Brendan P. Lucey

**Affiliations:** ^1^ Department of Psychological & Brain Sciences Washington University in St. Louis St Louis Missouri USA; ^2^ Department of Biostatistics Washington University in St. Louis St Louis Missouri USA; ^3^ Department of Neurology Washington University in St. Louis St Louis Missouri USA; ^4^ Knight Alzheimer Disease Research Center Washington University in St. Louis St Louis Missouri USA; ^5^ Department of Radiology Washington University in St. Louis St Louis Missouri USA; ^6^ Center On Biological Rhythms and Sleep Washington University in St. Louis St Louis Missouri USA

**Keywords:** Alzheimer's disease, amyloid, single‐channel electroencephalography, sleep, sleep logs

## Abstract

**INTRODUCTION:**

Sleep disturbances are associated with Alzheimer's disease (AD) pathology and cognitive symptoms, but few studies have longitudinally assessed sleep, AD biomarkers, and cognition. Understanding how sleep changes over time in older adults with and without AD pathology is crucial for appropriately designing longitudinal studies of aging and AD.

**METHODS:**

Sleep was measured over ≈3.5 years in older adults using self‐reported questionnaires and an at‐home single‐channel electroencephalography (scEEG) device. Participants also underwent amyloid imaging and cognitive testing. Longitudinal change of multiple sleep parameters was determined and sample sizes estimated for future clinical trials using sleep as an outcome.

**RESULTS:**

In both amyloid groups, EEG spectral power measures demonstrated minimal longitudinal change, whereas self‐reported and sleep parameters such as sleep efficiency demonstrated greater variability over time.

**DISCUSSION:**

This study characterized how sleep parameters change in older adults with and without AD pathology, offering important guidance for future longitudinal studies targeting sleep and neurodegeneration.

**Highlights:**

- Low variability in electroencephalography (EEG) spectral power metrics, including delta, theta, and alpha power.
- Greater variability in self‐report sleep metrics, including self‐reported time to fall asleep and EEG‐derived metrics such as sleep duration and N2 sleep.
- Understanding the variability in sleep measures over time will offer important guidance for designing future longitudinal studies targeting sleep and neurodegeneration.

## 1 BACKGROUND

Alzheimer's disease (AD) has grown increasingly prevalent as a major cause of dementia and death in elderly individuals.^1^, ^2^ AD has a long preclinical phase that precedes clinical diagnosis, where pathology begins to develop but cognition remains unimpaired.^3^, ^4^, ^5^ Changes in blood, cerebrospinal fluid (CSF), and positron emission tomography (PET) AD biomarkers provide early indication of AD pathology before clinical symptoms arise. Levels of amyloid beta (Aβ) plaques, captured by blood, CSF, or PET biomarkers, begin to develop a decade or more before the onset of symptoms.^6^ These pathological changes in the brain are expected to precede other larger alterations due to AD, including neuronal injury, cell death, and ultimately clinical changes, including memory and cognitive decline.^7^ The preclinical stage serves as a crucial window for intervention to target progression from asymptomatic to symptomatic AD.^8^

Sleep disturbances are commonly seen in both the “preclinical” asymptomatic period of AD, when AD pathology is accumulating without cognitive dysfunction, and in symptomatic AD.^9^ Alterations in sleep efficiency, duration, and time spent in certain sleep stages while in the preclinical phase have been shown to be associated with cognitive decline and Aβ deposition.^10^, ^11^ Disrupted sleep affects 25% of people with mild dementia and up to 50% of those with moderate or severe dementia.^12^ It was previously thought that sleep changes were due to underlying AD pathology, but there has been mounting evidence that sleep disturbances also contribute to an increased risk of AD.^13^ This bidirectional relationship is poorly understood due to a lack of research on sleep, AD biomarkers, and cognitive assessments collected longitudinally.

There are a multitude of reported sleep‐monitoring paradigms, including polysomnography and self‐reports. For example, self‐report data—such as sleep diaries and surveys like the Epworth Sleepiness Scale and Pittsburgh Sleep Quality Index—are often used to measure sleep quality and disturbances due to ease of implementation.^14^‐^‐^ ^16^ Alternatively, multiple sleep parameters measured by single‐channel electroencephalography (scEEG) are comparable to those produced from polysomnography, including time spent in different sleep stages and EEG spectral power.^17^ Each approach produces numerous individual measurements that vary in sensitivity and specificity, leading to different studies potentially yielding varying results based on the chosen measurement methods.^18^, ^19^ Change over time serves as the foundation for assessing the sensitivity and predictability of a measure.^20^ Therefore, it is critical to understand how sleep metrics change over time when planning clinical research studies.

Currently there is a lack of research examining longitudinal sleep measures, AD biomarkers, and cognitive data. Many studies have relied on cross‐sectional data for sleep measures, which capture only a single timepoint for sleep's role in AD progression.^11^ This study sought to examine longitudinal sleep changes in a cohort of cognitively unimpaired amyloid‐negative and amyloid‐positive individuals to inform future sleep studies in older adults, particularly in the setting of AD.

## 2 METHODS

### 2.1 Participants

Sixty community‐living participants enrolled in longitudinal studies at the Charles F. and Joanne Knight Alzheimer's Disease Research Center (ADRC) at Washington University in St. Louis were included in the study. All participants underwent annual standardized clinical and cognitive assessments by a clinician. For inclusion in this study, participants were required to have: (1) two separate study visits with six nights of sleep monitoring from an scEEG device and sleep logs; (2) an amyloid PET scan within 2 years of sleep monitoring; and (3) a cognitive assessment of their Clinical Dementia Rating (CDR) within 1 year of sleep monitoring. Age, sex, race, years of education, and medical history were also collected. This study was approved by the Washington University Institutional Review Board. Each participant provided written informed consent and was compensated for their participation.

### 2.2 Sleep monitoring

Sleep was monitored at two timepoints with an average 3.49‐year difference between visits. At each timepoint, sleep was assessed up to six nights at home using an scEEG device (Sleep Profiler, Advanced Brain Monitoring, Carlsbad, CA). Bed and rise times were confirmed with sleep logs and actigraphy. The scEEG device was worn on the forehead and recorded 256 samples per second from three frontal sensors placed at approximately AF7, AF8, and Fpz. Only the AF7‐AF8 channel was used for scoring sleep stages in 30 s epochs in Polysmith (Nihon Kohden, Tokyo, Japan) by registered polysomnographic technologists using modified American Academy of Sleep Medicine (AASM) criteria.^17^ Summary sleep measures from the scored scEEG epochs were calculated using Polysmith. Nights were excluded if >10% of the recording was artifactual and/or if the bed and rise times did not match the sleep log and/or actigraphy. Participants needed a minimum of two nights that met this criterion to be included in the analyses. Average total sleep duration, wake after sleep onset, sleep efficiency, rapid eye movement (REM) onset latency, and time in different sleep stages (non‐REM (NREM) sleep Stages 1, 2, 3, and REM) were calculated for each study night and used in all analyses. Sleep efficiency was calculated based on the lights‐off and lights‐on times for the scEEG studies. Sleep stages and measures derived from the scEEG are referred to as scEEG‐derived metrics throughout the article.

**RESEARCH IN CONTEXT**

1. **Systematic review**: We used PubMed to perform a literature search for studies examining sleep in the context of Alzheimer's disease. The majority of prior work has examined sleep cross‐sectionally, and there is limited knowledge on the test–retest reliability of widely utilized sleep measures in amyloid‐negative and amyloid‐positive older adults.
2. **Interpretation**: Objective sleep measures, primarily electroencephalography spectral power, reflected the lowest longitudinal variability over ≈3.5 years. Objective sleep measures provide greater reliability when studying sleep longitudinally when compared to self‐report measures in both amyloid‐negative and amyloid‐positive individuals.
3. **Future directions**: Future studies are needed to examine longitudinal sleep in those who convert from amyloid‐negative to amyloid‐positive.

Spectral analysis was performed to quantify EEG power within different frequency bins. As described previously,^17^ a band‐pass (two‐way least‐squares finite impulse response) filter between 0.5 and 40 Hz was applied to the scEEG data. Spectral analysis was performed in consecutive 5 s epochs (Welch method, Hamming window, no overlap). Power bands during NREM sleep were calculated by averaging the power in the frequency bins of 0.5–1.0 Hz for <1 Hz slow‐wave activity (SWA), 1.0–4.5 Hz for SWA, 4–8 Hz for theta, 8–12 Hz for alpha, 12–16 Hz for spindles, 15–25 Hz for beta, and 25–50 Hz for gamma waves. To semi‐automatically remove artifactual epochs, power in the 20–30 and 0.5–4.5 Hz bands for each electrode across all epochs of a recording was displayed. The operator (B.P.L.) then selected a threshold between the 95% and 99.5% threshold of power to remove artifactual epochs. Each frequency range was calculated for each single‐channel EEG study using MATLAB (MathWorks, Natick, MA), and the average NREM frequency range was used in the analysis.

As mentioned previously, participants completed daily logs and questionnaires. These included the Epworth Sleepiness Scale,^15^ the Insomnia Severity Index,^21^ daytime napping, time to fall asleep, and self‐reported sleep duration. Data derived from these reports are referred to as self‐reported data in all analyses. Participants also underwent one night of home sleep apnea testing as described previously (HSAT; Alice PDx, Philips Respironics, Inc., Murrysville, PA).^22^ The Alice PDx is a type III HSAT device that monitors oxygen saturation (SpO_2_) and pulse rate from an oximeter finger probe, nasal pressure‐based airflow monitor and thermistor, thoracic and abdominal effort via inductance plethysmography, and body position. A minimum of 4 h of artifact‐free recording was obtained for all participants, and participants not meeting this criterion were asked to repeat monitoring. Respiratory events were scored by registered polysomnographic technologists using AASM criteria^23^ and were reviewed by a board‐certified sleep medicine physician (B.P.L.). Hypopneas were scored using 4% oxygen desaturation criteria. The apnea‐hypopnea index (AHI) was calculated per hour of monitoring time for each participant. Participants using positive airway pressure (PAP) therapy or dental devices were asked to use them as usual during the HSAT.

### 2.3 Neuroimaging

Participants underwent PET imaging with amyloid tracers. Amyloid PET imaging was performed using Pittsburgh compound B (PiB) or [18F]AV‐45 (florbetapir) and data from the 30 to 60 and 50‐ to 70‐min post‐injection window, respectively. To obtain a global measure of amyloid burden, the magnetic resonance imaging (MRI)–free PET processing pipeline was run for all amyloid PET scans.^24^ The pipeline uses Statistical Parametric Mapping 12's (SPM12)N1 normalization module to non‐linearly warp the PET data into alignment with a pre‐defined template PET image. The PET data are then spatially smoothed using a Gaussian filter to a full width at half maximum (FWHM) of 8 mm. A mean cortical standard uptake value ratio (MCSUVR) is calculated from the resulting image using the standard Centiloid (CL) cortical volume of interest (VOI) with the whole cerebellum VOI as the reference region.^25^ These SUVRs are then converted to the Centiloid scale. MCSUVRs from the MRI‐free PET pipeline are non‐partial volume corrected. Participants were considered amyloid‐negative by PET if their Centiloid value was less than 19 at Visit 1.^26^

### 2.4 Cognitive assessments

Cognitive assessment protocols were consistent with the National Alzheimer's Coordinating Center (NACC) Uniform Data Set (UDS).^27^ The CDR was performed at each clinical assessment to determine the presence or absence of dementia, and when present, its severity. CDR is used in longitudinal studies and clinical trials for staging dementia in general and in dementia due to AD, with an overall score ranging from 0 to 3 and a sum of boxes (SB) ranging from 0 to 18.^28^, ^29^ Participants included in the study were classified as either cognitively unimpaired (CDR = 0, *n* = 55) or mildly impaired (CDR = 0.5, *n* = 4; CDR = 1, *n* = 1). We did not assess the differences between CDR = 0 and CDR >0 due to the limited number of CDR >0 participants.

### 2.5 Statistical analysis

To determine if the sleep metrics changed over time, intraclass correlation coefficients (ICC) were calculated. The R Package *irr* was used.^30^ General linear models were used to determine the longitudinal change of sleep parameters computed at the subject level as the ratio of the difference in each parameter over the time difference in years (i.e., rate of change per year), whereas controlling for Centiloid value, age, sex, race, and if participants were positive for one apolipoprotein E (*APOE*) ε4 allele (ApoE4+). We also conducted power calculations for two types of studies: (1) a one‐sample longitudinal observational study monitoring change from baseline with participants serving as their own controls; and (2) a two‐arm clinical trial with both treated and untreated groups. For the one‐sample power analysis, we computed the mean and SD of this estimated rate of change for each sleep parameter and then determined the sample size necessary to detect longitudinal change in the sleep measures. The power analysis was based on a one‐sample *t*‐test with a two‐sided alternative hypothesis. We defined the detectable rate of change as 0.5, 0.8, 1, 1.2, 1.5, and 2 times the observed rate of change, targeting 80% power, and determined the corresponding sample sizes. For the two‐sample power analysis, we used a two‐sample *t*‐test on the rate of change with a two‐sided alternative hypothesis. We treated the observed data as representing the control group and assumed the treatment group would exhibit a rate of change (i.e., change/year) equal to 1%, 3%, 5%, and 7% of the baseline value. For each assumed effect size, we calculated the required sample size to achieve 80% power at a significance level of 0.05. The power analyses were conducted using R 4.4.0.^31^

### 2.6 Data and material availability

All of the data that support the findings of this study are available from the corresponding author upon reasonable request or via the Washington University in St. Louis Charles F. and Joanne Knight Alzheimer's Disease Research Center's data request portal.^32^ All code associated with this analysis is freely available from the corresponding author upon reasonable request.

## 3 RESULTS

### 3.1 Participant characteristics

Table 1 shows the breakdown of participants between groups and characteristics associated with sleep. This includes sleep apnea and related metrics (AHI and average saturated oxygen percentage), and medical history including insomnia, restless leg syndrome, and depression. The number of participants who reported using a sleep medication is also shown. Participants were listed as on a sleep medication if they were taking at least one of the following medications: benzodiazepine receptor agonists (zolpidem, zaleplon, eszopiclone), benzodiazepines (triazolam, temazepam, alprazolam), suvorexant, ramelteon, gabapentin, dopamine agonists (ropinirole, pramipexole, rotigotine), doxepin, antihistamines, antidepressants, and narcotics. In order to be more representative of the general population, participants were not excluded if they had a sleep‐related disorder or used sleep medications.^33^ Comparisons between groups and visits were made using a two‐sample *t*‐test for age, visit interval, AHI, and average SaO_2_%. All other comparisons were made using a chi‐square test. Amyloid Centiloid value was the only metric that showed a significant difference between amyloid groups at each visit. Notably, there were no significant differences in Centiloid value within amyloid groups and between visits (amyloid‐negative *p*‐value = 0.416, amyloid‐positive *p*‐value = 0.119). This aligned with the other demographics and sleep characteristics, as there were no significant differences within or between amyloid groups. No participants changed amyloid groups or CDR status between visits. Differences between amyloid groups for all sleep metrics are shown in Tables S1–S3.

**TABLE 1:** Participant characteristics.

Demographics	Amyloid negative	Amyloid positive	*p*‐value^*^	Overall
Sample size	*n* = 37	*n* = 23		*n* = 60
Age (at first visit)				
Mean (SD)	72.48 (4.16)	73.66 (4.08)	0.740 (0.962)	72.96 (4.13)
Median [Min, Max]	71.96 [64.0, 83.4]	73.76 [65.7, 82.3]		72.50 [64.1, 83.4]
Sex, *n* (%)				
Female	19 (51.4%)	13 (56.5%)	0.901 (0.976)	32 (53.3%)
Male	18 (48.6%)	10 (43.5%)		28 (46.7%)
Race, *n* (%)				
Black	6 (16.2%)	2 (8.7%)	0.405 (0.962)	8 (13.3%)
White	31 (83.8%)	21 (91.3%)		52 (86.7%)
CDR = 0	34 (91.9%)	21 (91.3%)	0.946 (0.976)	55 (91.6%)
CDR >0	3 (0.08%)	2 (0.09%)		5 (0.08%)
Centiloid value				
Visit 1, mean (SD)	5.00 (6.10)	45.88 (24.08)	7.59e‐14 (6.07e‐13)	
Visit interval, years				
Mean (SD)	3.45 (0.79)	3.54 (0.87)	0.688 (0.962)	3.49 (0.814)
Median [Min, Max]	3.30 [2.48, 5.80]	3.35 [2.45, 6.01]		3.32 [2.45, 6.01]
Sleep apnea (AHI ≥5)				
Visit 1	6 (16.2%)	5 (21.7%)	0.726 (0.962)	11 (18.3%)
Visit 2	14 (37.8%)	9 (39.1%)		23 (38.3%)
AHI (events/h)				
Visit 1, mean (SD)	8.75 (7.65)	7.17 (9.44)	0.616 (0.962)	–
Visit 2, mean (SD)	8.48 (8.89)	4.62 (7.5)	0.154 (0.810)	–
Average SaO_2_ (%)				
Visit 1, mean (SD)	92.91 (1.85)	93.39 (1.58)	0.640 (0.962)	‐
Visit 2, mean (SD)	92.97 (1.51)	93.78 (1.52)	0.053 (0.695)	‐
Insomnia				
Visit 1	2 (5.4%)	4 (17.4%)	1.00 (1.00)	6 (10.0%)
Visit 2	2 (5.4%)	4 (17.4%)		6 (10.0%)
Restless leg syndrome, *n* (%)				
Visit 1	4 (10.8%)	2 (8.7%)	0.533 (0.962)	6 (10.0%)
Visit 2	4 (10.8%)	4 (17.4%)		8 (13.3%)
Depression, *n* (%)				
Visit 1	4 (10.8%)	1 (4.3%)	0.187 (0.810)	5 (8.3%)
Visit 2	1 (2.7%)	2 (8.7%)		3 (5.0%)
# PTs using sleep medication (%)				
Visit 1	9 (24.3%)	7 (30.4%)	0.870 (0.976)	16 (26.7%)
Visit 2	8 (21.6%)	7 (30.4%)		15 (25.0%)

Abbreviations: AHI, Apnea‐Hypopnea Index; CDR, Clinical Dementia Rating; Max, maximum; Min, minimum; PTs, participants; SaO_2_, arterial oxygen saturation; SD, standard deviation.

* Included both original and adjusted for multiple comparison *p*‐values: original (adjusted). Bolded values = *p*‐value < 0.05.

### 3.2 Longitudinal variability of sleep metrics

**FIGURE 1:**
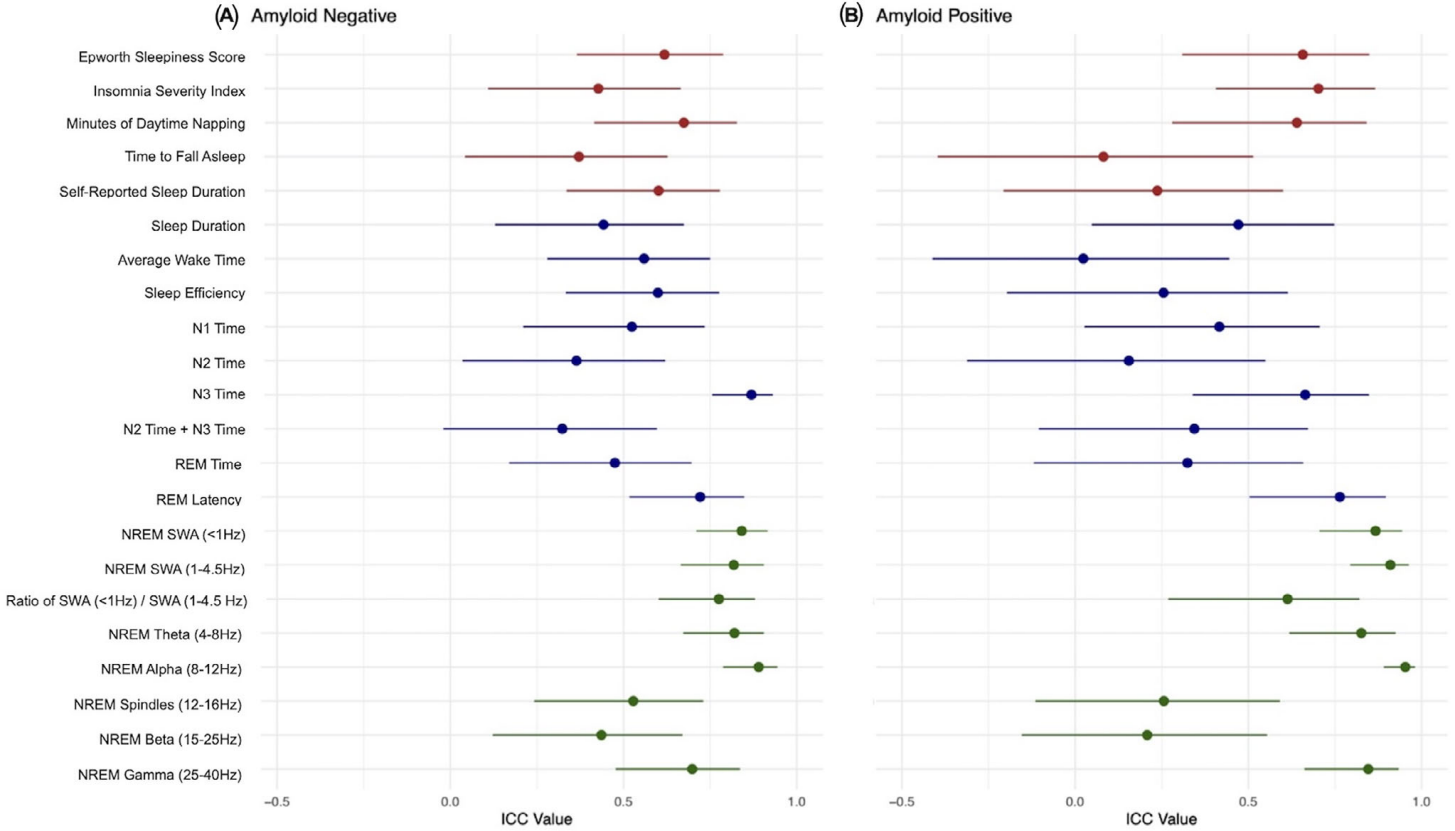
Intraclass Correlation Coefficients. The ICC for each sleep metric is shown. (A) ICC values for the amyloid‐negative group. (B) ICC values for the amyloid‐positive group. Error bars represent the confidence interval around the ICC value. Variables labeled represent self‐reported (red), scEEG‐derived (blue), and EEG spectral power measures (green). EEG, electroencephalography; Hz, hertz; ICC, intraclass correlation coefficient; N1, NREM Stage 1 sleep; N2, NREM Stage 2 sleep; N3, NREM Stage 3 sleep; NREM, non‐rapid eye movement; REM, rapid eye movement; scEEG: single‐channel EEG; SWA, slow‐wave activity.

Figure 1 shows the ICC values for each sleep metric in amyloid‐negative (Figure 1A) and amyloid‐positive (Figure 1B) individuals. The ICC is a measure of agreement over time, and the interpretation is as follows: below 0.5 indicates poor agreement, 0.5–0.75 represents moderate agreement, 0.75–0.9 shows good agreement, and above 0.9 reflects excellent agreement.^34^

For both amyloid groups, the EEG spectral power metrics had the lowest longitudinal variability and the greatest agreement between visits. In the amyloid‐negative group, EEG spectral power reflected the highest ICC values (mean ICC = 0.719) compared to the scEEG‐based measures (mean = 0.542) and self‐report data (mean = 0.538). A similar trend is seen in amyloid‐positive individuals: EEG spectral power measures also reflected the greatest ICC values (mean ICC = 0.695), compared to self‐report (mean ICC = 0.463) and scEEG‐derived metrics (mean ICC = 0.367).

Within both amyloid groups, certain sleep metrics stood out in their level of agreement. In the scEEG‐derived metrics, average NREM Stage 3 (N3) sleep time showed the lowest longitudinal variability (amyloid‐negative ICC = 0.869; amyloid‐positive ICC = 0.664). This aligns with the ICC values derived from EEG spectral power measures, considering that N3 sleep is characterized by slow‐wave sleep and increased SWA. Slow‐wave sleep measures, which included average NREM SWA (<1 Hz), NREM SWA (1–4.5 Hz), and the ratio of NREM SWA (<1 Hz) to NREM SWA (1–4.5 Hz), all showed good to excellent agreement (amyloid‐negative ICC = 0.841, 0.818, and 0.775, respectively; amyloid‐positive ICC = 0.867, 0.910, and 0.613, respectively). Average NREM theta (4–8 Hz) and NREM alpha (8–12 Hz) also reflected EEG spectral power bands with good to excellent longitudinal agreement (amyloid‐negative ICC = 0.820 and 0.890, respectively; amyloid‐positive ICC = 0.826 and 0.953, respectively), which is consistent between amyloid groups. As for the self‐report sleep metrics, no metric surpassed the threshold of ICC = 0.75, but daytime napping and self‐reported sleep duration approached it. Of note, the variability of self‐reported sleep duration was markedly higher in the amyloid‐positive group compared to the amyloid‐negative group (amyloid‐negative ICC = 0.601; amyloid‐positive ICC = 0.237).

To better gauge how these sleep metrics perform on the individual level, longitudinal change for one sleep metric was graphed within each sleep metric category (Figures S1–S3) and reflects the longitudinal change of self‐reported minutes of daytime napping, sleep duration, and NREM slow‐wave activity. These metrics were chosen out of interest due to their current use in studies examining the role of sleep in AD.^35^, ^36^, ^37^, ^38^ The average deviation from Visit 1 (depicted by the dashed line) indicated that these measures showed minimal change over time, on average. On the individual level, there is a distribution of deviations, with the majority reflecting minor changes between visits.

### 3.3 Longitudinal variability adjusting for potential confounders

Because multiple factors could affect sleep over time, generalized linear modeling was used to determine the annual change of each sleep metric after adjusting for Centiloid, age, sex, race, and ApoE4+ status. The annual rate of change was defined as the metric at Visit 2 minus the metric at Visit 1 divided by the time between visits in years. Table 2 includes model outputs from self‐report measures, Table 3 shows scEEG‐derived measures, and Table 4 includes EEG spectral power measures. Results from the linear models indicated low longitudinal change across sleep metrics, even after accounting for Centiloid, sex, age, race, and ApoE4+ status. None of the covariates were significant predicators (*p* > 0.05) of the annualized rate of change per sleep metric after correcting for multiple comparisons. These findings were consistent with the findings of the ICC analyses.

**TABLE 2:** Annual rate of change of self‐report metrics adjusting for potential confounders.

Sleep variable annualized rate of change	Covariate	Estimate	SE	*t*‐statistic	*p*‐value	Adjusted *p*‐value
**Epworth Sleepiness Scale**	Centiloid value	1.9 × 10⁻⁴	0.01	0.03	0.979	1.00
	Age	0.02	0.05	0.44	0.659	1.00
	Sex	−0.44	0.39	−1.13	0.265	1.00
	Race	−0.38	0.56	−0.68	0.502	1.00
	*APOE* ε4 status	0.17	0.40	0.43	0.676	1.00
**Insomnia Severity Index**	Centiloid value	4.3 × 10⁻^3^	0.01	0.73	0.470	1.00
	Age	0.03	0.04	0.79	0.435	1.00
	Sex	−0.39	0.31	−1.28	0.206	1.00
	Race	0.55	0.44	1.25	0.216	1.00
	*APOE* ε4 status	0.18	0.31	0.58	0.565	1.00
**Daytime napping**	Centiloid value	−0.02	0.03	−0.61	0.543	1.00
	Age	0.04	0.20	0.18	0.861	1.00
	Sex	−1.93	1.64	−1.17	0.246	1.00
	Race	−0.31	2.35	−0.13	0.897	1.00
	*APOE* ε4 status	−0.81	1.68	−0.48	0.632	1.00
**Time to fall asleep**	Centiloid value	−0.02	0.04	−0.48	0.635	1.00
	Age	−0.32	0.22	−1.47	0.147	1.00
	Sex	−2.80	1.77	−1.58	0.119	1.00
	Race	0.39	2.71	0.14	0.886	1.00
	*APOE* ε4 status	−1.37	1.82	−0.75	0.454	1.00
**Self‐reported sleep duration**	Centiloid value	4.0 × 10⁻^3^	1.6 × 10⁻^3^	2.54	0.014	0.35
	Age	4.8 × 10⁻^3^	0.01	0.48	0.631	1.00
	Sex	4.7 × 10⁻⁴	0.08	0.01	0.995	1.00
	Race	0.14	0.12	1.21	0.230	1.00
	*APOE* ε4 status	−0.05	0.08	−0.63	0.525	1.00

Abbreviations: *APOE* ε4, apolipoprotein E ε4 allele4; SE, standard error.

Adjusted *p*‐values are after Bonferroni correction for multiple comparisons.

**TABLE 3:** Annual rate of change of scEEG‐derived measures adjusting for potential confounders.

Sleep variable annualized rate of change	Covariate	Estimate	SE	*t*‐statistic	*p*‐value	Adjusted *p*‐value
**Sleep duration**	Centiloid value	−0.04	0.10	−0.40	0.694	1.00
	Age	0.46	0.62	0.74	0.465	1.00
	Sex	−2.05	5.07	−0.41	0.687	1.00
	Race	1.28	7.25	0.18	0.861	1.00
	*APOE* ε4 status	−2.00	5.18	−0.39	0.700	1.00
**Average wake time**	Centiloid value	0.17	0.85	0.20	0.731	1.00
	Age	0.17	0.85	0.20	0.746	1.00
	Sex	1.87	1.31	1.43	0.750	1.00
	Race	5.84	2.31	2.53	0.621	1.00
	*APOE* ε4 status	2.00	0.03	66.67	0.637	1.00
**Sleep efficiency**	Centiloid value	−0.02	0.02	−1.38	0.172	1.00
	Age	−0.05	0.11	−0.45	0.654	1.00
	Sex	−0.05	0.88	−0.06	0.953	1.00
	Race	−0.67	1.26	−0.53	0.599	1.00
	*APOE* ε4 status	−0.62	0.90	−0.69	0.490	1.00
**Average N1 time**	Centiloid value	0.01	0.02	0.40	0.690	1.00
	Age	0.02	0.15	0.12	0.908	1.00
	Sex	0.18	1.20	0.15	0.884	1.00
	Race	0.16	1.71	0.09	0.926	1.00
	*APOE* ε4 status	−0.31	1.22	−0.25	0.800	1.00
**Average N2 time**	Centiloid value	0.08	0.12	0.69	0.493	1.00
	Age	−0.18	0.76	−0.24	0.810	1.00
	Sex	−9.81	6.17	−1.59	0.118	1.00
	Race	−1.79	8.82	−0.20	0.840	1.00
	*APOE* ε4 status	−1.28	6.31	−0.20	0.840	1.00
**Average N3 time**	Centiloid value	−2.5 × 10⁻⁴	0.02	−0.02	0.988	1.00
	Age	−0.22	0.10	−2.15	0.037	1.00
	Sex	0.92	0.85	1.08	0.285	1.00
	Race	0.35	1.22	0.29	0.776	1.00
	*APOE* ε4 status	−0.46	0.87	−0.53	0.595	1.00
Average N2+N3 time	Centiloid value	0.082	0.120	0.683	0.497	1.00
	Age	−0.408	0.765	−0.533	0.596	1.00
	Sex	−8.89	6.22	−1.43	0.159	1.00
	Race	−1.44	8.90	−0.162	0.872	1.00
	*APOE* ε4 status	−1.74	6.36	−0.274	0.785	1.00
**Average REM time**	Centiloid value	0.00	0.04	0.10	0.918	1.00
	Age	0.34	0.27	1.29	0.202	1.00
	Sex	−1.16	2.17	−0.53	0.595	1.00
	Race	2.99	3.10	0.96	0.340	1.00
	*APOE* ε4 status	−3.21	2.21	−1.45	0.153	1.00
**REM latency**	Centiloid value	−0.04	0.07	−0.49	0.629	1.00
	Age	−0.08	0.48	−0.16	0.872	1.00
	Sex	1.60	3.89	0.41	0.683	1.00
	Race	−2.36	5.57	−0.42	0.673	1.00
	*APOE* ε4 status	1.27	3.98	0.32	0.752	1.00

Abbreviations: *APOE* ε4, apolipoprotein E ε4 allele4; N1, nonrapid eye movement sleep stage 1; N2, nonrapid eye movement sleep stage 2; N3, nonrapid eye movement sleep stage 3; REM, rapid eye movement; SE, standard error.

Adjusted *p*‐values are after Bonferroni correction for multiple comparisons.

**TABLE 4:** Annual rate of change of EEG spectral power measures adjusting for potential confounders.

Sleep variable annual rate of change	Covariate	Estimate	SE	*t*‐statistic	*p*‐value	Adjusted *p*‐value
Average NREM SWA (<1 Hz)	Centiloid value	−0.04	0.04	−0.92	0.361	1.00
	Age	0.10	0.29	0.35	0.730	1.00
	Sex	1.85	2.33	0.80	0.430	1.00
	Race	−1.68	3.33	−0.51	0.615	1.00
	*APOE* ε4 status	0.64	2.38	0.27	0.788	1.00
Average NREM SWA (1–4.5 Hz)	Centiloid value	−0.01	0.01	−1.13	0.264	1.00
	Age	0.06	0.07	0.92	0.361	1.00
	Sex	0.17	0.53	0.33	0.743	1.00
	Race	−0.31	0.76	−0.41	0.686	1.00
	*APOE* ε4 status	0.25	0.54	0.46	0.640	1.00
Ratio of <1 Hz SWA/1–4.5 Hz SWA	Centiloid value	2.4 × 10⁻⁴	6.1 × 10⁻⁴	0.398	0.692	1.00
	Age	−2.9 × 10⁻^3^	0.289	0.790	0.433	1.00
	Sex	0.180	0.032	0.570	0.571	1.00
	Race	0.028	0.046	0.613	0.543	1.00
	*APOE* ε4 status	0.052	0.326	1.58	0.119	1.00
Average NREM theta (4–8 Hz)	Centiloid value	−1.6 × 10⁻^3^	1.0 × 10⁻^3^	−1.55	0.127	1.00
	Age	0.01	0.01	1.70	0.095	1.00
	Sex	−0.02	0.05	−0.31	0.759	1.00
	Race	−0.06	0.08	−0.81	0.421	1.00
	*APOE* ε4 status	0.05	0.06	0.83	0.377	1.00
Average NREM alpha (8–12 Hz)	Centiloid value	−7.3 × 10⁻⁵	4.9 × 10⁻⁴	−0.15	0.881	1.00
	Age	4.0 × 10⁻^3^	3.1 × 10⁻^3^	1.27	0.209	1.00
	Sex	−0.04	0.03	−1.55	0.127	1.00
	Race	−0.04	0.04	−1.04	0.302	1.00
	*APOE* ε4 status	−0.03	0.03	−1.00	0.331	1.00
Average NREM spindles (12–16 Hz)	Centiloid value	−0.01	2.2 × 10⁻^3^	−2.43	0.019	0.76
	Age	−3.7 × 10⁻^3^	0.01	−0.26	0.793	1.00
	Sex	−0.07	0.11	−0.59	0.558	1.00
	Race	−0.48	0.16	−2.96	0.005	0.2
	*APOE* ε4 status	−0.03	0.12	−0.25	0.772	1.00
Average NREM beta (15–25 Hz)	Centiloid value	−0.01	2.1 × 10⁻^3^	−2.58	0.013	0.52
	Age	−4.7 × 10⁻^3^	0.01	−0.36	0.721	1.00
	Sex	−0.05	0.11	−0.46	0.650	1.00
	Race	−0.46	0.15	−2.98	0.004	0.16
	*APOE* ε4 status	−0.02	0.11	−0.18	0.882	1.00
Average NREM gamma (25–40 Hz)	Centiloid value	−2.4 × 10⁻⁵	3.8 × 10⁻⁵	−0.63	0.531	1.00
	Age	2.9 × 10⁻⁴	2.5 × 10⁻⁴	1.19	0.240	1.00
	Sex	−4.4 × 10⁻⁴	2.0 × 10⁻^3^	−0.22	0.828	1.00
	Race	7.5 × 10⁻⁴	2.9 × 10⁻^3^	0.26	0.796	1.00
	*APOE* ε4 status	2.7 × 10⁻^3^	2.1 × 10⁻^3^	0.00	0.191	1.00

Abbreviations: *APOE* ε4, apolipoprotein E ε4 allele; Hz, hertz; NREM, non‐rapid eye movement; SE, standard error; SWA, slow‐wave activity.

Adjusted *p*‐values are after Bonferroni correction for multiple comparisons.

### 3.4 Sample size estimates

**FIGURE 2:**
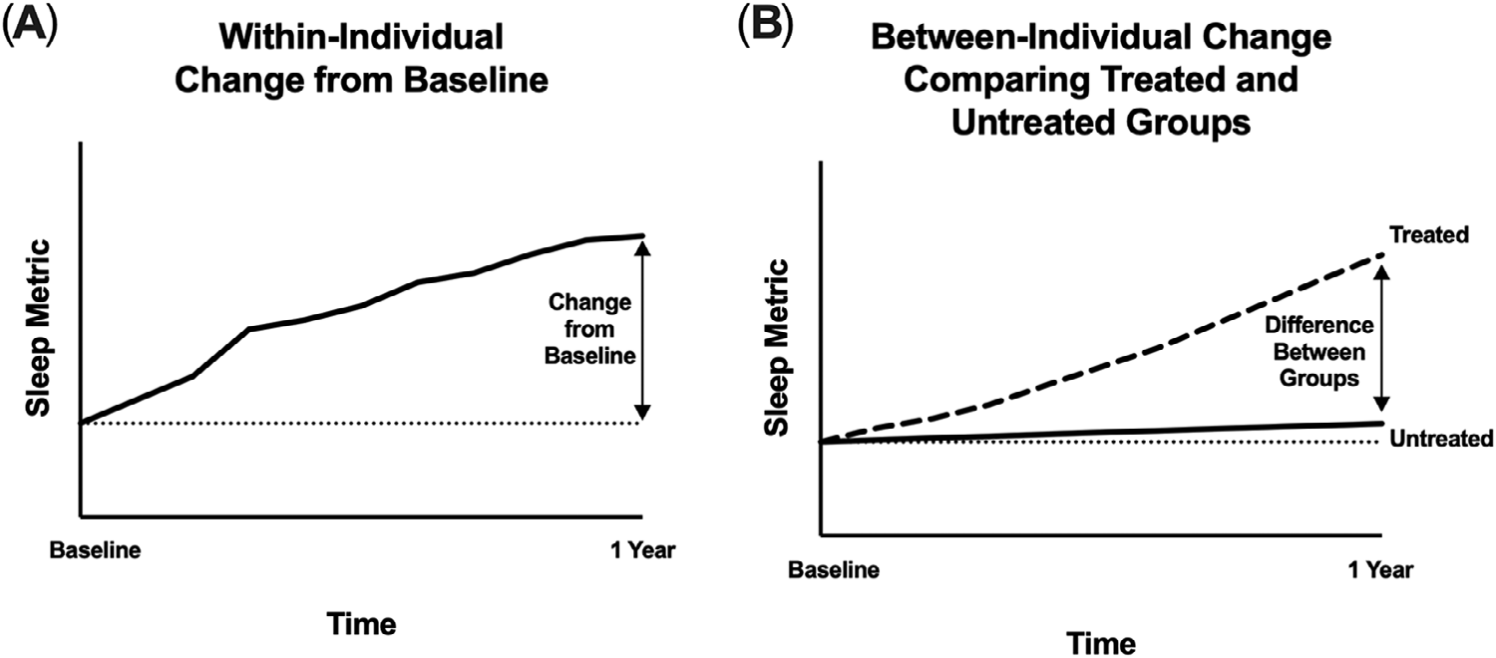
Outcomes of Potential Clinical Study Designs. (A) Example of the outcomes from a study measuring one‐sample within‐individual change in a sleep metric from baseline with participants serving as their own controls. The within‐individual change from baseline at 1 year is shown. (B) Example of the outcomes from a two‐arm clinical trial measuring between‐individual change comparing treated and untreated groups. The untreated group shows a low rate of change at 1 year, similar to our findings. The treated group increases the sleep metric, and the between‐individual group differences at 1 year are shown. The x‐axis shows time from the baseline study start to 1 year. The y‐axis indicates the sleep metric. The horizontal dotted line shows no change from baseline.

To assess how these findings may be applicable to different clinical study designs, power analyses were conducted to estimate the sample sizes necessary to detect both within‐individual and between‐individual longitudinal change of different sleep metrics depending on the study design. For a one‐sample (within‐individual) longitudinal observational study monitoring change from baseline with participants serving as their own controls, the detectable rate of change was used to calculate the sample size needed to detect the following proportions of the observed rate of change: 0.5, 0.8, 1.0, 1.2, 1.5, and 2.0. Tables S4–S6 show the sample sizes needed for using self‐reported, scEEG‐derived, and EEG power sleep metrics. Each table includes the average rate of change per year and the SD of the rate of change per metric. To test a two‐arm clinical trial (between‐individual) comparing treated and untreated groups, we used the baseline value, observed rate of change, and SD of the rate of change to calculate the sample sizes needed to measure group differences of 1%, 3%, 5%, and 7% (Tables S7–S9). Figure 2 shows examples of these two types of studies.

In Table 5, we show example sample size calculations for both studies of within‐individual (one‐sample) change from baseline and between‐individual (two‐arm clinical trial) change, comparing treated and untreated groups with self‐reported minutes of daytime napping, sleep duration measured by scEEG, and average 1–4.5 Hz NREM SWA. For the within‐individual study design, we used 5% change from baseline as a minimum clinically meaningful change in the sleep metrics. Sleep metrics from Visit 1 in Tables S1–S3 were used for baseline values. For sleep duration and average NREM SWA, the sample size necessary to detect a significant change at 80% power is <40 participants. In comparison, the self‐reported daytime napping measure calls for 574 amyloid‐negative participants and 120 amyloid‐positive participants, respectively. In the between‐individual study design, the 5% and 7% change between groups are shown in Table 5, and all measures can be differentiated with sample sizes between 5 and 156 participants per group.

**TABLE 5:** Example sample size calculations.

*Within‐individual sample sizes to detect a 5% change from baseline*								
	Amyloid negative				Amyloid positive			
Sleep metric	Baseline value	5% change from baseline	SD of rate of change	Sample size (*n*)	Baseline value	5% change from baseline	SD of rate of change	Sample size (*n*)
Self‐reported daytime napping (min)	14.04	0.70	5.99	574	23.12	1.16	4.54	120
Sleep duration (min)	371.6	18.58	19.5	7	367.4	18.37	16.2	6
Average NREM SWA (1–4.5 Hz)	19.15	0.96	2.06	36	19.09	0.95	1.75	27

*Between‐individual sample sizes comparing treated and untreated groups*												
	Amyloid negative						Amyloid positive					
					Sample size per group (*n*)						Sample size per group (*n*)	
Sleep metric	Observed rate of change	SD of rate of change	Baseline value	Observed rate of change/baseline	5% change	7% change	Observed rate of change	SD of rate of change	Baseline value	Observed rate of change/baseline	5% change	7% change
Self‐reported daytime napping (min)	−0.017	0.268	7.250	−0.23%	9	6	−4.37x10^−2^	0.251	7.346	−0.06%	9	5
Sleep duration (min)	5.245	19.46	370.1	1.42%	35	15	−0.9685	16.24	375.7	−0.26%	12	7
Average NREM SWA (1–4.5 Hz)	0.2607	2.060	18.33	1.42%	156	65	−6.00x10^−3^	1.749	20.26	−0.03%	48	25

Abbreviations: Hz, hertz; *n* = sample size, min, minutes; NREM, non‐rapid eye movement; SD, standard deviation; SWA, slow‐wave activity.

## 4 DISCUSSION

Sleep disturbances have been associated with numerous disease states including AD, Parkinson's disease, major mental illnesses such as depression and anxiety, as well as cardiovascular disease and obesity.^39^, ^40^, ^41^, ^42^ Despite evidence supporting sleep's role in the onset and progression of certain diseases, there are few studies that follow sleep longitudinally while also collecting longitudinal cognitive and AD biomarker data. This study aimed to fill that gap by examining self‐reported sleep measures, scEEG‐derived sleep metrics, and EEG spectral power in a cohort of cognitively unimpaired to mildly impaired amyloid‐negative and amyloid‐positive individuals over the span of ≈3.5 years. The longitudinal change of sleep metrics within each amyloid group was estimated using an ICC, and findings were confirmed using general linear modeling that adjusted for the presence of covariates that may influence longitudinal change.

We observed that EEG spectral power measures had the least longitudinal change over time in both amyloid‐negative and amyloid‐positive individuals. EEG power measures from <1 to 12 Hz showed the greatest stability. This is consistent with prior findings in healthy individuals that EEG spectral power had a strong correlation (*r* = 0.84) over a 12‐ to 16‐week interval.^43^ In comparison, the scEEG‐derived measures and self‐reported sleep data demonstrated relatively greater longitudinal change over time, with metrics ranging from low to moderate change between visits. However, certain metrics stood out for their greater stability within these categories. Minutes of daytime napping, average N3 time, and REM latency each exhibited lower longitudinal change (amyloid‐negative ICC = 0.674, 0.869, and 0.721, respectively; amyloid‐positive ICC = 0.640, 0.664, and 0.764, respectively). The annual rate of change of each sleep measure was also not associated with Centiloid value, sex, age, race, or ApoE4+ status.

Overall, the evaluated sleep metrics reflected generally low rates of change across the ≈3.5‐year interval. A possible explanation for these findings is the lack of significant change in amyloid Centiloid values or cognitive status between visits. For example, neither participant's overall CDR scores nor amyloid PET Centiloid values changed significantly over the follow‐up period. We hypothesize that the lack of change observed in the different sleep metrics may be due to the lack of change in AD symptoms or pathology. If so, this suggests that relatively small sample sizes with longitudinal sleep, AD biomarker, and cognitive measures are needed to establish if sleep changes over time as amyloid plaques accumulate or cognition worsens. Alternatively, our study may be underpowered to detect changes in sleep over ≈3.5 years in cognitively unimpaired and mildly impaired amyloid‐negative and amyloid‐positive older adults. Future studies with larger sample sizes that capture changes in amyloid and/or cognitive status are needed to establish if sleep changes with AD progression and if sleep measures may serve as a functional biomarker in early AD.

As a result of these small rates of change combined with small variances, the sample sizes for within‐individual change from baseline were very large for some measures to detect even *k* = 2.0 change (Tables S4–S6). However, such a small rate of change may not be considered clinically meaningful. For example, sleep duration in the amyloid‐positive group has an average rate of change of −0.969 min/year. A 50% change in this metric is equal to 0.485 min/year and requires 8831 participants to detect this change with 80% power. Although the sample size in this scenario is markedly large, the example highlights the importance of choosing a clinically meaningful effect for a study outcome. A randomized clinical trial of suvorexant to treat insomnia in patients with mild‐to‐moderate AD found a significant increase in sleep duration of 28 min,^44^ strongly supporting that this is a clinically meaningful increase. Our data show that only three cognitively unimpaired amyloid‐positive participants are needed to see a 30 min/year change in sleep duration in 1 year using the one‐sample within‐individual study design. This example underscores the critical importance of selecting the appropriate study outcome measure when choosing an experimental study design.

The study findings suggest that the majority of sleep metrics require sample sizes too large to use as primary outcomes in randomized controlled trials. However, these sample sizes, which may also be adjusted based on hypothesized effect size, may be more appropriate for longitudinal observational studies or as a covariate to follow an intervention when AD pathology and cognitive function may change. An example of how our findings may be applicable to a “real world” study would be to assess sleep longitudinally in AD patients receiving anti‐amyloid immunotherapy (e.g., lecanemab, donanemab). A potential sleep metric to follow is 1–4.5 Hz NREM SWA, a measure of sleep homeostasis that has been proposed to track both AD pathology and cognitive performance.^45^ If we assume a longitudinal change similar to what we found in amyloid‐positive older adults, only 27 amyloid‐positive individuals would be needed to adequately power a study with an outcome of a 5% change in NREM SWA from baseline in patients treated with anti‐amyloid therapy using the one‐sample within‐individual study design (Figure 2A, Table 5). To detect 5% or 7% differences in NREM SWA between treated and untreated groups, a greater number of participants would be required than in the one‐sample study example, and a different sleep metric may serve as a better outcome measure depending on the study hypothesis and expected effect size. The above examples are for illustrative purposes and do not account for other factors that may impact sleep such as underlying cognitive impairment and other medical comorbidities.

The strengths of this study included well‐characterized participants and the large amount of sleep data acquired using different methods. Participants were assessed for longitudinal AD biomarkers, standardized cognitive assessments, and sleep monitored with both EEG and self‐reported methods. A limitation of this study was the lack of participants who changed amyloid or CDR status. A future direction will be to examine the longitudinal change of sleep metrics in those who convert in cognitive status or amyloid status, as the study is still ongoing. Although we have found that sleep measures derived from scEEG are comparable to those from polysomnography,^17^ it is important to note that there are important differences, including low agreement for N1. In addition, these findings apply only to an older adult cohort. Participant ages ranged from 64 to 83 years (Table 1). There are unique changes in sleep‐related architecture that occur in young adults as well as in mid‐life that are not captured in this study.^46^

In summary, this study serves as a resource to inform future longitudinal sleep studies in the setting of AD. EEG spectral power measures showed the least longitudinal change and required generally smaller sample sizes to detect longitudinal changes in sleep. Future studies of sleep and AD will need to select the sleep metric and study design to be appropriately powered to detect meaningful effect sizes.

## CONFLICT OF INTEREST STATEMENT

Brendan P. Lucey receives consulting fees from Eisai, Eli Lilly, and the Weston Family Foundation; serves on data safety and monitoring boards for Eli Lilly; serves on the scientific advisory board for Beacon Biosignals; and receives compensation as a scientific advisor to Applied Cognition. He receives drug/matched placebo from Merck for a clinical trial funded by a private foundation and drug/matched placebo from Eisai for a clinical trial funded by the National Institute on Aging (NIA). David M. Holtzman is a cofounder of C2N Diagnostics; he has an equity ownership interest and may receive income based on technology licensed by Washington University to C2N Diagnostics. He reports serving on the scientific advisory board of C2N Diagnostics, Denali, Genentech, Cajal Neuroscience, and Switch Therapeutics, and consults for Roche. John C. Morris is funded by NIH grants # P30 AG066444, P01AG003991, and P01AG026276. Neither Dr. Morris nor his family owns stock or has equity interest (outside of mutual funds or other externally directed accounts) in any pharmaceutical or biotechnology company.

Authors Taylor J. Pedersen, Ruijin Lu, Cristina Toedebusch, Ashley Hess, Rachel Richardson, Allyson Quigley, Carling G. Robinson, Chengjie Xiong, and Brian A. Gordon have no disclosures. Any author disclosures are available in the Supporting Information.

## CONSENT STATEMENT

This study was approved by the Washington University Institutional Review Board. Each participant provided written informed consent and were compensated for their participation.

## Supporting information



Supporting Information

Supporting Information
