# Supplementary material for: Variability of longitudinal sleep monitoring in amyloid‐negative and amyloid‐positive cognitively unimpaired and mildly impaired older adults
Source: Alzheimers Dement. 2025 Oct 8;21(10):e70761. doi: 10.1002/alz.70761 (PMC12505026; doi:10.1002/alz.70761)
Supplement: Supplementary file 1 — Supporting Information [file ALZ-21-e70761-s002.docx]

**Supplemental Figures**


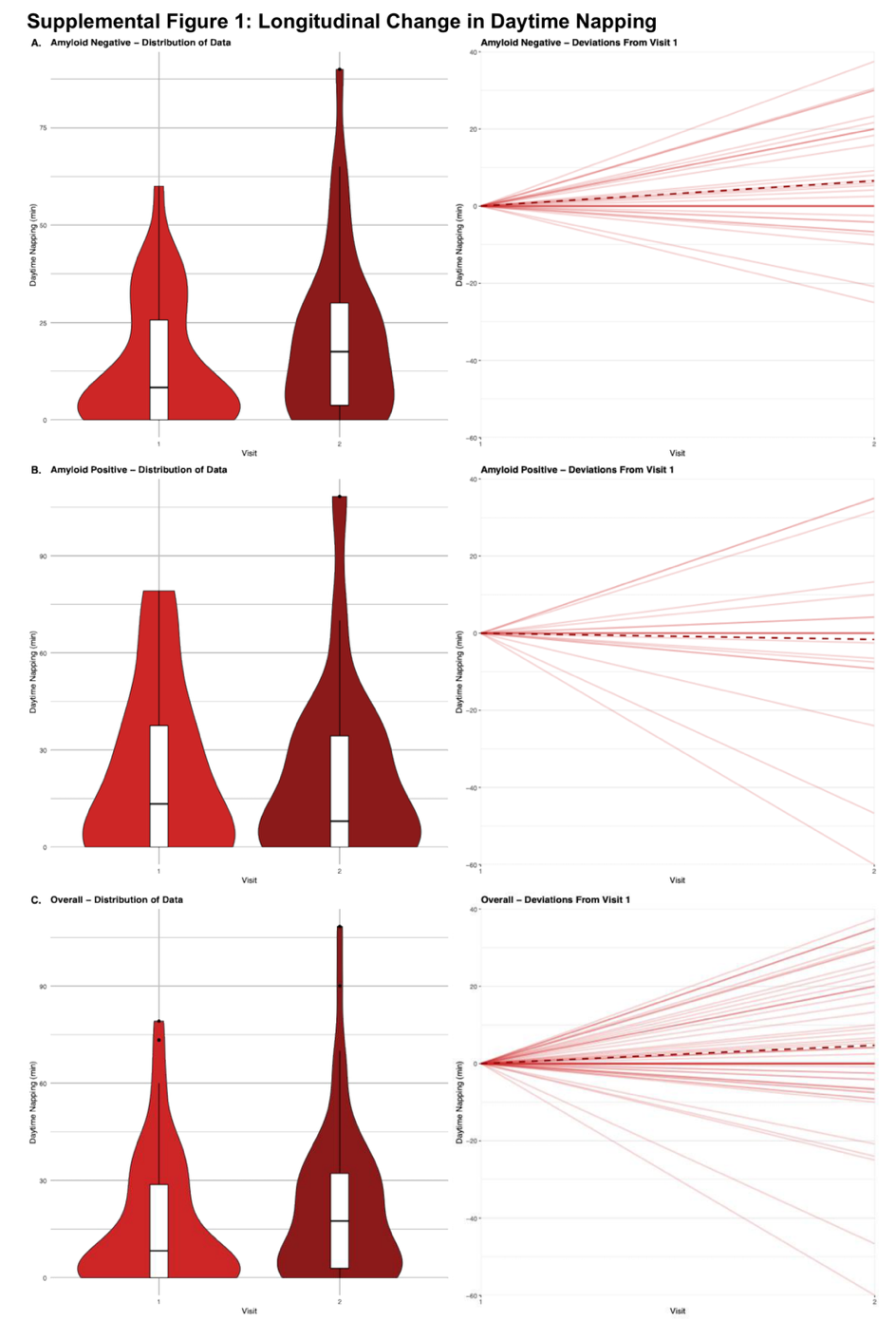


Supplemental Figure 1: Longitudinal Change in Minutes of Daytime Napping

The distribution of longitudinal self-reported minutes of daytime napping data and individual trajectories. A) Trajectories of daytime napping in amyloid-negative participants are shown as violin plots and individual change from baseline. B) Trajectories of daytime napping in amyloid-positive participants are shown as violin plots and individual change from baseline. C) Trajectories of daytime napping in all participants are shown as violin plots and individual change from baseline.

**
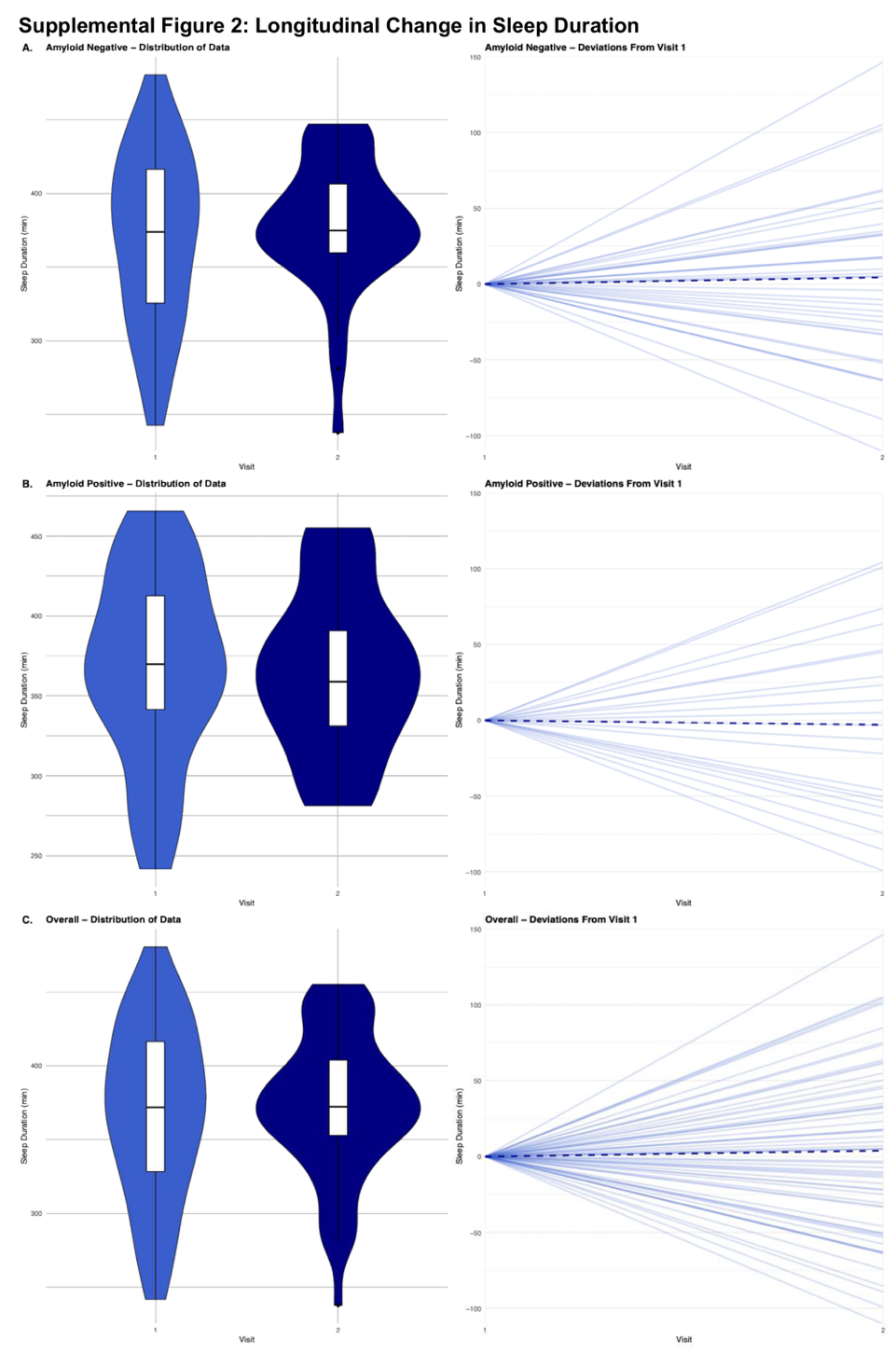
**

Supplemental Figure 2: Longitudinal Change in Sleep Duration

The distribution of longitudinal single-channel EEG-derived sleep duration and individual trajectories. A) Trajectories of sleep duration in amyloid-negative participants are shown as violin plots and individual change from baseline. B) Trajectories of sleep duration in amyloid-positive participants are shown as violin plots and individual change from baseline. C) Trajectories of sleep duration in all participants are shown as violin plots and individual change from baseline.


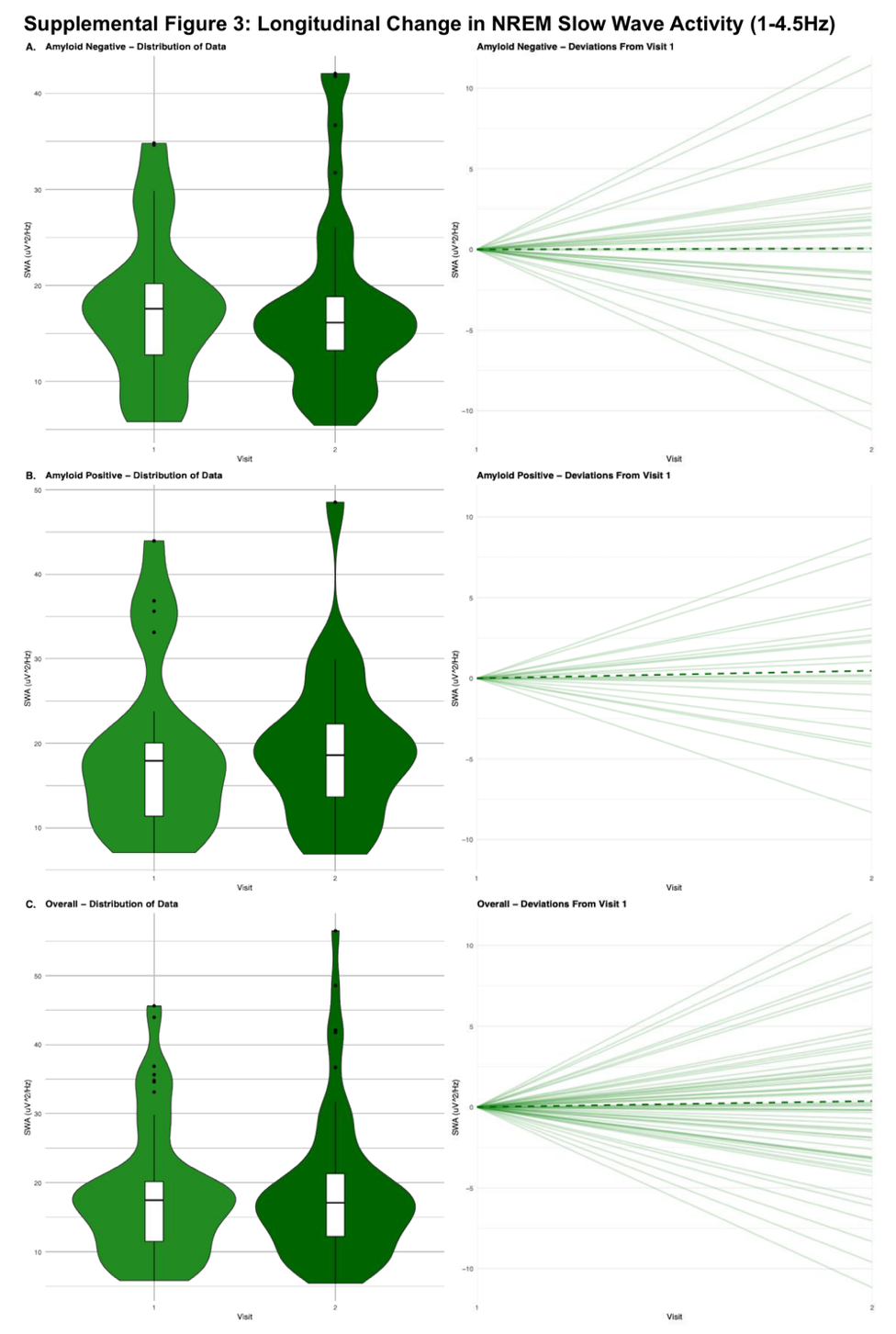


Supplemental Figure 3: Longitudinal Change in 1-4.5 Hz Non-Rapid Eye Movement Slow Wave Activity

The distribution of longitudinal 1-4.5 Hz Non-Rapid Eye Movement Slow Wave Activity (NREM SWA) and individual trajectories. A) Trajectories of NREM SWA in amyloid-negative participants are shown as violin plots and individual change from baseline. B) Trajectories of NREM SWA in amyloid-positive participants are shown as violin plots and individual change from baseline. C) Trajectories of sleep duration in all participants are shown as violin plots and individual change from baseline.

| **Supplemental Table 1: Differences in Longitudinal Self-Report Metrics Between Amyloid Groups** | | | | | | | | |
| --- | --- | --- | --- | --- | --- | --- | --- | --- |
|  | **Amyloid Negative** | | | **Amyloid Positive** | | |  | |
|  | **Visit 1** | **Visit 2** |  | **Visit 1** | **Visit 2** |  | **Between Group Differences** | |
| **Sleep Metric (units)** | **Mean (SD)** | **Mean (SD)** | **p-value between visits*** | **Mean (SD)** | **Mean (SD)** | **p-value between visits*** | **p-value**  **(Visit 1)*** | **p-value**  **(Visit 2)*** |
| **Epworth Sleepiness Scale (0-24)** | 8.49 (3.86) | 7.91 (4.79) | 0.404 (0.904) | 7.30 (5.36) | 7.75 (6.87) | 0.620 (0.904) | 0.273 (0.904) | 0.847 (0.943) |
| **Insomnia Severity Index (0-28)** | 8.80 (3.64) | 8.74 (4.67) | 0.602 (0.904) | 8.24 (3.70) | 9.00 (4.34) | 0.284 (0.904) | 0.225 (0.904) | 0.633 (0.904) |
| **Daytime Napping (min)** | 14.04 (16.3) | 20.56 (21.68) | 0.095 (0.904) | 23.12 (26.06) | 21.52 (28.06) | 0.837 (0.943) | 0.303 (0.904) | 0.943 (0.943) |
| **Time to Fall Asleep (min)** | 22.85 (21.04) | 26.10 (20.56) | 0.3499 (0.904) | 20.49 (14.54) | 18.27 (10.56) | 0.428 (0.904) | 0.873 (0.943) | **0.047 (0.904)** |
| **Self-Reported Sleep Duration (min)** | 436.20 (51.60) | 430.20 (51.60) | 0.929 (0.943) | 424.80 (67.20) | 440.40 (66.00) | 0.501 (0.904) | 0.605 (0.904) | 0.727 (0.943) |
| Abbreviations: **SD** = standard deviation, **min** = minutes.  *****Included both original and adjusted for multiple comparison p-values: original (adjusted).  **Bolded** values represent a p-value <0.05 prior to correction for multiple comparisons. | | | | | | | | |

**Supplemental Tables**

The mean and standard deviation (SD) of each sleep metric were calculated within each amyloid group and at each study visit (**Supplemental Tables 1-3**). **Supplemental Table 1** shows the mean and SD of each self-reported sleep metric. **Supplemental Table 2** reports the mean and SD of scEEG-derived measurements within amyloid groups and visits. Similarly, mean and SD were calculated for the all-night EEG spectral power metrics **(Supplemental Table 3).** Comparisons between amyloid groups and visits were made using a paired t-test. There were no significant differences between visits or groups across sleep metrics after correction for multiple comparisons.

| **Supplemental Table 2: Differences in Longitudinal scEEG-Derived Metrics Between Amyloid Groups** | | | | | | | | |
| --- | --- | --- | --- | --- | --- | --- | --- | --- |
|  | **Amyloid Negative** | | | **Amyloid Positive** | | |  | |
|  | **Visit 1** | **Visit 2** |  | **Visit 1** | **Visit 2** |  | **Between Group Differences** | |
| **Sleep Metric (units)** | **Mean (SD)** | **Mean (SD)** | **p-value between visits*** | **Mean (SD)** | **Mean (SD)** | **p-value between visits*** | **p-value**  **(Visit 1)*** | **p-value**  **(Visit 2)*** |
| **Sleep Duration (min)** | 371.6 (58.81) | 375.99 (44.98) | 0.516 (0.993) | 367.38 (63.21) | 364.38 (53.67) | 0.870 (0.993) | 0.903 (0.993) | 0.337 (0.993) |
| **Average Wake Time (min)** | 85.08 (51.83) | 89.31(52.62) | 0.848 (0.993) | 70.99 (33.32) | 81.36 (47.59) | 0.527 (0.993) | 0.196 (0.993) | 0.486 (0.993) |
| **Sleep Efficiency (%)** | 77.97 (10.0) | 78.26 (9.78) | 0.701 (0.993) | 80.34 (7.69) | 78.79 (9.38) | 0.616 (0.993) | 0.250 (0.993) | 0.838 (0.993) |
| **Average N1 Time (min)** | 33.94 (14.28) | 27.48 (12.98) | **0.019 (0.680)** | 31.48 (10.77) | 24.36 (16.72) | 0.366 (0.993) | 0.273 (0.993) | 0.987 (0.995) |
| **Average N2 Time (min)** | 215.92 (44.54) | 211.95 (44.48) | 0.949 (0.995) | 204.25 (44.04) | 203.75 (50.8) | 0.995 (0.995) | 0.418 (0.993) | 0.425 (0.993) |
| **Average N3 Time (min)** | 8.8 (16.22) | 10.96 (18.23) | 0.441 (0.993) | 9.91 (12.98) | 12.11 (17.93) | 0.808 (0.993) | 0.692 (0.993) | 0.910 (0.993) |
| **Average N2 + N3 Time (min)** | 236.49 (54.96) | 236.38 (39.71) | 0.655 (0.993) | 232.56 (55.18) | 238.6 (46.33) | 0.764 (0.993) | 0.906 (0.993) | 0.857 (0.993) |
| **Average REM Time (min)** | 78.23 (25.03) | 80.47 (22.67) | 0.439 (0.993) | 82.14 (19.68) | 86.32 (24.64) | 0.565 (0.993) | 0.492 (0.993) | 0.622 (0.993) |
| **REM Latency (min)** | 100.02 (51.91) | 110.25 (61.05) | 0.439 (0.993) | 88.26 (40.52) | 86.21 (46.74) | 0.738 (0.993) | 0.659 (0.993) | 0.549 (0.993) |
| Abbreviations: **SD** = standard deviation, **min** = minutes.  *****Included both original and adjusted for multiple comparison p-values: original (adjusted).  **Bolded** values represent a p-value <0.05 prior to correction for multiple comparisons. | | | | | | | | |

| **Supplemental Table 3: Differences in Longitudinal EEG Spectral Power Between Amyloid Groups** | | | | | | | | |
| --- | --- | --- | --- | --- | --- | --- | --- | --- |
|  | **Amyloid Negative** | | | **Amyloid Positive** | | |  | |
|  | **Visit 1** | **Visit 2** |  | **Visit 1** | **Visit 2** |  | **Between Group Differences** | |
| **Sleep Metric (μV^2^/Hz)** | **Mean (SD)** | **Mean (SD)** | **p-value between visits*** | **Mean (SD)** | **Mean (SD)** | **p-value between visits*** | **p-value**  **(Visit 1)*** | **p-value**  **(Visit 2)*** |
| **Average NREM SWA (<1Hz)** | 62.63 (44.02) | 69.16 (69.4) | 0.447 (0.970) | 59.8 (34.74) | 65.02 (35.49) | 0.947 (0.970) | 0.953 (0.970) | 0.511 (0.970) |
| **Average NREM SWA (1-4.5Hz)** | 19.15 (11.57) | 20.07 (16.53) | 0.544 (0.970) | 19.09 (10.27) | 19.56 (9.37) | 0.734 (0.970) | 0.709 (0.970) | 0.565 (0.970) |
| **Ratio of (<1Hz) SWA / (1-4.5Hz) SWA** | 3.21 (0.54) | 3.3 (0.61) | 0.505 (0.970) | 3.1 (0.56) | 3.3 (0.65) | 0.216 (0.970) | 0.398 (0.970) | 0.970 (0.970) |
| **Average NREM Theta (4-8Hz)** | 2.64 (1.12) | 2.7 (1.3) | 0.522 (0.970) | 2.65 (1.86) | 2.7 (1.02) | 0.632 (0.970) | 0.575 (0.970) | 0.582 (0.970) |
| **Average NREM Alpha (8-12Hz)** | 1.33 (0.79) | 1.22 (0.67) | 0.927 (0.970) | 1.26 (0.71) | 1.2 (0.83) | 0.361 (0.970) | 0.667 (0.970) | 0.575 (0.970) |
| **Average NREM Spindles (12-16Hz)** | 1.8 (0.98) | 1.64 (1.23) | 0.950 (0.970) | 2.65 (1.86) | 1.69 (1.09) | 0.425 (0.970) | 0.239 (0.970) | 0.838 (0.970) |
| **Average NREM Beta (15-25Hz)** | 1.39 (0.81) | 1.29 (1.06) | 0.797 (0.970) | 2.14 (1.78) | 1.23 (0.9) | **0.018 (0.622)** | **0.039 (0.622)** | 0.683 (0.970) |
| **Average NREM Gamma (25-40Hz)** | 0.06 (0.03) | 0.06 (0.04) | 0.772 (0.970) | 0.06 (0.03) | 0.06 (0.03) | 0.387 (0.970) | 0.422 (0.970) | 0.690 (0.970) |
| Abbreviations: **SD** = standard deviation, **min** = minutes.  *****Included both original and adjusted for multiple comparison p-values: original (adjusted).  **Bolded** values represent a p-value <0.05 prior to correction for multiple comparisons. | | | | | | | | |

| **Supplemental Table 4: Within-Individual Sample Size Estimates of Self-Report Metrics** | | | | | | | | | | | | | | | | | | |
| --- | --- | --- | --- | --- | --- | --- | --- | --- | --- | --- | --- | --- | --- | --- | --- | --- | --- | --- |
|  | **Amyloid Negative** | | | | | | | | | **Amyloid Positive** | | | | | | | | |
| **Sleep Metric** | **Rate of Change (per year)** | **SD of Rate of Change** | **Detectable Rate of Change (0.5)** | **n (k=0.5)** | **n (k=0.8)** | **n (k=1)** | **n (k=1.2)** | **n (k=0.1.5)** | **n (k=2.0)** | **Rate of Change (per year)** | **SD of Rate of Change** | **Detectable Rate of Change (0.5)** | **n (k=0.5)** | **n (k=0.8)** | **n**  **(k=1.0)** | **n (k=1.2)** | **n (k=1.5)** | **n (k=2.0)** |
| **Epworth Sleepiness Scale (0-24)** | -0.048 | 1.063 | 0.024 | 15708 | 6138 | 3929 | 2729 | 1748 | 984 | -0.008 | 1.15 | 0.005 | 662627 | 258840 | 165659 | 115041 | 73627 | 41416 |
| **Insomnia Severity Index (0-28)** | 0.186 | 1.30 | 0.093 | 1548 | 606 | 389 | 271 | 174 | 99 | 0.029 | 0.820 | 0.015 | 24339 | 9509 | 6087 | 4228 | 2706 | 1523 |
| **Daytime Napping (min)** | 1.83 | 5.99 | 0.914 | 340 | 134 | 87 | 61 | 40 | 24 | 0.308 | 4.54 | 0.154 | 6806 | 2660 | 1703 | 1184 | 758 | 428 |
| **Time to Fall Asleep (min)** | 0.309 | 7.37 | 0.154 | 17868 | 6981 | 4469 | 3104 | 1987 | 1119 | 0.771 | 6.20 | 0.390 | 2029 | 794 | 509 | 354 | 228 | 129 |
| **Self-Reported Sleep Duration (hrs)** | -0.017 | 0.268 | 0.008 | 8278 | 3235 | 2071 | 1439 | 922 | 520 | -0.004 | 0.251 | 0.002 | 103522 | 40440 | 25882 | 17975 | 11505 | 6472 |
| Abbreviations: **SD** = standard deviation, **min** = minutes, **n** = sample size, **k** = proportion for the detectable rate of change | | | | | | | | | | | | | | | | | | |

| **Supplemental Table 5: Within-Individual Sample Size Estimates of scEEG-Derived Measures** | | | | | | | | | | | | | | | | | | |
| --- | --- | --- | --- | --- | --- | --- | --- | --- | --- | --- | --- | --- | --- | --- | --- | --- | --- | --- |
|  | **Amyloid Negative** | | | | | | | | | **Amyloid Positive** | | | | | | | | |
| **Sleep Metric** | **Rate of Change (per year)** | **SD of Rate of Change** | **Detectable Rate of Change (0.5)** | **n (k=0.5)** | **n (k=0.8)** | **n (k=1.0)** | **n (k=1.2)** | **n (k=0.1.5)** | **n (k=2.0)** | **Rate of Change (per year)** | **SD of Rate of Change** | **Detectable Rate of Change (0.5)** | **n (k=0.5)** | **n (k=0.8)** | **n (k=1.0)** | **n (k=1.2)** | **n (k=0.1.5)** | **n (k=2.0)** |
| **Sleep Duration (min)** | 5.24 | 19.5 | 2.62 | 435 | 171 | 111 | 78 | 51 | 30 | -0.969 | 16.2 | 0.484 | 8831 | 3451 | 2210 | 1535 | 983 | 554 |
| **Average Wake Time (min)** | -1.83 | 18.0 | 0.917 | 3036 | 1188 | 761 | 529 | 340 | 192 | -1.43 | 17.2 | 0.712 | 4572 | 1787 | 1145 | 796 | 510 | 288 |
| **Sleep Efficiency (%)** | 0.830 | 3.04 | 0.415 | 422 | 166 | 107 | 75 | 49 | 29 | 0.216 | 3.08 | 0.063 | 18719 | 7313 | 4681 | 3252 | 2082 | 1172 |
| **Average N1 Time (min)** | -1.11 | 4.39 | 0.552 | 497 | 196 | 126 | 88 | 57 | 33 | -2.65 | 3.25 | 1.33 | 50 | 21 | 14 | 11 | 8 | 6 |
| **Average N2 Time (min)** | 3.27 | 15.2 | 1.63 | 686 | 269 | 173 | 121 | 78 | 45 | -0.621 | 12.9 | 0.310 | 13483 | 5268 | 3373 | 2343 | 1500 | 845 |
| **Average N3 Time (min)** | 0.731 | 2.59 | 0.365 | 397 | 156 | 101 | 71 | 46 | 27 | 0.461 | 3.84 | 0.231 | 2182 | 854 | 547 | 381 | 245 | 139 |
| **Average N2 + N3 Time (min)** | 4.00 | 15.2 | 2.00 | 459 | 181 | 116 | 82 | 53 | 31 | -0.159 | 13.9 | 0.080 | 237892 | 92928 | 59475 | 41303 | 26435 | 14870 |
| **Average REM Time (min)** | 2.21 | 7.61 | 1.10 | 376 | 148 | 96 | 67 | 44 | 26 | 1.84 | 7.80 | 0.921 | 565 | 222 | 143 | 100 | 65 | 38 |
| **REM Latency (min)** | 0.881 | 15.4 | 0.441 | 9646 | 3769 | 2413 | 1677 | 1074 | 605 | -0.280 | 11.3 | 0.138 | 52832 | 20639 | 13210 | 9174 | 5872 | 3304 |
| Abbreviations: **SD** = standard deviation, **REM** = rapid eye movement, **min** = minutes, **n** = sample size, **k** = proportion for the detectable rate of change | | | | | | | | | | | | | | | | | | |

| **Supplemental Table 6: Within-Individual Sample Size Estimate of EEG Spectral Power** | | | | | | | | | | | | | | | | | | |
| --- | --- | --- | --- | --- | --- | --- | --- | --- | --- | --- | --- | --- | --- | --- | --- | --- | --- | --- |
|  | **Amyloid Negative** | | | | | | | | | **Amyloid Positive** | | | | | | | | |
| **Sleep Metric (μV^2^/Hz)** | **Rate of Change (per year)** | **SD of Rate of Change** | **Detectable Rate of Change (0.5)** | **n (k=0.5)** | **n (k=0.8)** | **n (k=1)** | **n (k=1.2)** | **n (k=0.1.5)** | **n (k=2.0)** | **Rate of Change (per year)** | **SD of Rate of Change** | **Detectable Rate of Change (0.5)** | **n (k=0.5)** | **n (k=0.8)** | **n**  **(k=1)** | **n (k=1.2)** | **n (k=0.1.5)** | **n (k=2.0)** |
| **Average NREM SWA (<1Hz)** | 1.99 | 9.43 | 0.997 | 704 | 277 | 178 | 124 | 80 | 46 | 1.01 | 7.22 | 0.506 | 1603 | 628 | 403 | 280 | 180 | 102 |
| **Average NREM SWA (1-4.5Hz)** | 0.261 | 2.06 | 0.130 | 1963 | 768 | 493 | 343 | 220 | 125 | -0.006 | 1.75 | 0.003 | 2664549 | 1040841 | 666139 | 462597 | 296063 | 166537 |
| **Ratio of (<1Hz) SWA / (1-4.5Hz) SWA** | 0.025 | 0.101 | 0.0125 | 513 | 202 | 130 | 91 | 59 | 34 | 0.037 | 0.145 | 0.019 | 491 | 193 | 125 | 87 | 57 | 33 |
| **Average NREM Theta (4-8Hz)** | 0.011 | 0.199 | 0.006 | 9804 | 3831 | 2453 | 1704 | 1092 | 615 | -0.010 | 0.206 | 0.005 | 13452 | 5256 | 3365 | 2337 | 1497 | 843 |
| **Average NREM Alpha (8-12Hz)** | -0.029 | 0.105 | 0.014 | 428 | 169 | 109 | 76 | 50 | 29 | -0.028 | 0.087 | 0.014 | 303 | 120 | 77 | 55 | 36 | 21 |
| **Average NREM Spindles (12-16Hz)** | -0.025 | 0.052 | 0.0127 | 135 | 54 | 35 | 25 | 17 | 11 | -0.010 | 0.045 | 0.005 | 659 | 259 | 167 | 116 | 75 | 44 |
| **Average NREM Beta (15-25Hz)** | -0.106 | 0.242 | 0.053 | 167 | 67 | 44 | 31 | 21 | 13 | -0.088 | 0.530 | 0.044 | 1141 | 447 | 287 | 200 | 129 | 74 |
| **Average NREM Gamma (25-40Hz)** | -0.001 | 0.007 | 0.001 | 984 | 386 | 248 | 173 | 112 | 64 | -0.002 | 0.007 | 0.001 | 428 | 169 | 109 | 76 | 50 | 29 |
| Abbreviations: **SD** = standard deviation, **NREM** = non-rapid eye movement, **SWA** = slow wave activity, **min** = minutes, **n** = sample size, **V** = volt, **Hz** = hertz, **k** = proportion for the detectable rate of change | | | | | | | | | | | | | | | | | | |

| **Supplemental Table 7: Between-Individual Sample Size Estimates of Self-Report Metrics Comparing Treated and Untreated Groups** | | | | | | | | | | | | | | | | |
| --- | --- | --- | --- | --- | --- | --- | --- | --- | --- | --- | --- | --- | --- | --- | --- | --- |
|  | **Amyloid Negative** | | | | | | | | **Amyloid Positive** | | | | | | | |
| **Sleep Variable** | **Observed Rate of Change** | **SD of Rate of Change** | **Baseline Value** | **Observed Rate of Change / Baseline** | **Sample Size (n)** | | | | **Observed Rate of Change** | **SD of Rate of Change** | **Baseline Value** | **Observed Rate of Change / Baseline** | **Sample Size (n)** | | | |
|  |  |  |  |  | **10% Change** | **30% Change** | **50% Change** | **70% Change** |  |  |  |  | **10% Change** | **30% Change** | **50% Change** | **70% Change** |
| **Epworth Sleepiness Scale (0-24)** | -0.048 | 1.063 | 7.618 | -0.62% | 1160 | 234 | 98 | 54 | -7.91x10^-3^ | 1.150 | 6.35 | -0.12% | 4069 | 528 | 197 | 103 |
| **Insomnia Severity Index (0-28)** | 0.186 | 1.303 | 8.676 | 2.14% | 2723 | 4799 | 435 | 151 | 0.029 | 0.820 | 8.35 | 0.35% | 3613 | 217 | 72 | 36 |
| **Daytime Napping (min)** | 1.828 | 5.992 | 11.38 | 16.05% | 193 | 257 | 357 | 532 | 0.308 | 4.540 | 20.96 | 1.47% | 33118 | 3156 | 593 | 243 |
| **Time to Fall Asleep (min)** | 0.390 | 7.370 | 26.86 | 1.15% | 524774 | 3454 | 799 | 347 | 0.771 | 6.199 | 17.84 | 4.33% | 1716 | 10798 | 41625 | 2651 |
| **Self-Reported Sleep Duration (hrs)** | -0.017 | 0.268 | 7.250 | -0.23% | 144 | 22 | 9 | 6 | -4.37x10^-2^ | 0.251 | 7.346 | -0.06% | 164 | 21 | 9 | 5 |
| Abbreviations: **SD** = standard deviations, **min** = minutes, **hrs** = hours. | | | | | | | | | | | | | | | | |

| **Supplemental Table 8: Between-Individual Sample Size Estimates of scEEG-Derived Measures Comparing Treated and Untreated Groups** | | | | | | | | | | | | | | | | |
| --- | --- | --- | --- | --- | --- | --- | --- | --- | --- | --- | --- | --- | --- | --- | --- | --- |
|  | **Amyloid Negative** | | | | | | | | **Amyloid Positive** | | | | | | | |
| **Sleep Variable** | **Observed Rate of Change** | **SD of Rate of Change** | **Baseline Value** | **Observed Rate of Change / Baseline** | **Sample Size (n)** | | | | **Observed Rate of Change** | **SD of Rate of Change** | **Baseline Value** | **Observed Rate of Change / Baseline** | **Sample Size (n)** | | | |
|  |  |  |  |  | **10% Change** | **30% Change** | **50% Change** | **70% Change** |  |  |  |  | **10% Change** | **30% Change** | **50% Change** | **70% Change** |
| **Sleep Duration (min)** | 5.245 | 19.46 | 370.1 | 1.42% | 2498 | 175 | 35 | 15 | -0.9685 | 16.24 | 375.7 | -0.26% | 187 | 29 | 12 | 7 |
| **Average Wake Time (min)** | -1.833 | 18.02 | 87.62 | -2.09% | 696 | 258 | 133 | 82 | -1.425 | 17.20 | 83.17 | -1.71% | 913 | 303 | 150 | 90 |
| **Sleep Efficiency (%)** | 0.8304 | 3.037 | 77.01 | 1.08% | 39828 | 68 | 17 | 9 | 0.1260 | 3.075 | 78.75 | 0.16% | 341 | 31 | 12 | 7 |
| **Average N1 Time (min)** | -1.106 | 4.391 | 35.46 | -3.12% | 143 | 66 | 38 | 25 | -2.651 | 3.252 | 30.89 | -8.58% | 20 | 15 | 11 | 9 |
| **Average N2 Time (min)** | 3.266 | 15.24 | 249.0 | 1.31% | 6061 | 208 | 45 | 20 | -0.6205 | 12.86 | 254.1 | -0.24% | 261 | 40 | 16 | 9 |
| **Average N3 Time (min)** | 0.7307 | 2.590 | 6.721 | 10.87% | 241 | 377 | 677 | 1555 | 0.4613 | 3.843 | 13.25 | 3.48% | 2147 | 57146 | 5721 | 1067 |
| **Average N2 + N3 Time (min)** | 3.997 | 15.23 | 255.7 | 1.56% | 1760 | 271 | 49 | 20 | -0.1593 | 13.86 | 267.3 | -0.06% | 377 | 47 | 18 | 10 |
| **Average REM Time (min)** | 2.205 | 7.609 | 80.88 | 2.73% | 468 | 18540 | 270 | 78 | 1.841 | 7.795 | 77.52 | 2.38% | 841 | 4069 | 232 | 76 |
| **REM Latency (min)** | 0.8811 | 15.44 | 95.82 | 0.92% | 629557 | 943 | 246 | 112 | -0.2756 | 11.31 | 95.71 | -0.29% | 1322 | 204 | 80 | 43 |
| Abbreviations: **SD** = standard deviations, **min** = minutes, **REM** = rapid eye movement. | | | | | | | | | | | | | | | | |

| **Supplemental Table 9: Between-Individual Sample Size Estimates of EEG Spectral Power Comparing Treated and Untreated Groups** | | | | | | | | | | | | | | | | |
| --- | --- | --- | --- | --- | --- | --- | --- | --- | --- | --- | --- | --- | --- | --- | --- | --- |
|  | **Amyloid Negative** | | | | | | | | **Amyloid Positive** | | | | | | | |
| **Sleep Metric (μV^2^/Hz)** | **Observed Rate of Change** | **SD of Rate of Change** | **Baseline Value** | **Observed Rate of Change / Baseline** | **Sample Size (n)** | | | | **Observed Rate of Change** | **SD of Rate of Change** | **Baseline Value** | **Observed Rate of Change / Baseline** | **Sample Size (n)** | | | |
|  |  |  |  |  | **10% Change** | **30% Change** | **50% Change** | **70% Change** |  |  |  |  | **10% Change** | **30% Change** | **50% Change** | **70% Change** |
| **Average NREM SWA (<1Hz)** | 1.994 | 9.427 | 59.22 | 3.37% | 712 | 29634 | 1492 | 303 | 1.012 | 7.222 | 63.22 | 1.60% | 5692 | 1047 | 179 | 72 |
| **Average NREM SWA (1-4.5Hz)** | 0.2607 | 2.060 | 18.33 | 1.42% | 11128 | 798 | 156 | 65 | -6.00x10^-3^ | 1.749 | 20.26 | -0.03% | 1105 | 129 | 48 | 25 |
| **Ratio of (<1Hz) SWA / (1-4.5Hz) SWA** | 0.02509 | 0.1011 | 3.152 | 0.80% | 3881 | 35 | 11 | 6 | 0.03746 | 0.1478 | 3.118 | 1.20% | 8685 | 110 | 26 | 12 |
| **Average NREM Theta (4-8Hz)** | 0.01125 | 0.1988 | 2.440 | 0.46% | 3594 | 163 | 52 | 26 | -9.95x10^-3^ | 0.2058 | 3.020 | -0.33% | 414 | 67 | 27 | 15 |
| **Average NREM Alpha (8-12Hz)** | -0.02858 | 0.1052 | 1.289 | -2.22% | 103 | 40 | 22 | 14 | -0.0282 | 0.0872 | 1.434 | -1.97% | 67 | 25 | 13 | 9 |
| **Average NREM Spindles (12-16Hz)** | -0.02537 | 0.05203 | 0.6142 | -4.13% | 44 | 24 | 15 | 11 | -9.93x10^-3^ | 0.0454 | 0.8568 | -1.16% | 96 | 27 | 13 | 8 |
| **Average NREM Beta (15-25Hz)** | -0.1056 | 0.2418 | 1.495 | -7.06% | 65 | 42 | 30 | 22 | -0.088 | 0.530 | 1.934 | -4.55% | 384 | 208 | 131 | 90 |
| **Average NREM Gamma (25-40Hz)** | -1.245x10^-3^ | 6.963x10^-3^ | 0.05835 | -2.13% | 229 | 86 | 45 | 28 | -1.87x10^-3^ | 6.89x10^-3^ | 0.06827 | -2.74% | 116 | 50 | 28 | 18 |
| Abbreviations: **SD** = standard deviations, **min** = minutes, **REM** = rapid eye movement, **SWA** = slow wave activity, **V =** volt, **Hz** = hertz. | | | | | | | | | | | | | | | | |
